# Cardiac tissue engineering: current state-of-the-art materials, cells and tissue formation

**DOI:** 10.1590/S1679-45082018RB4538

**Published:** 2018-09-11

**Authors:** Isabella Caroline Pereira Rodrigues, Andreas Kaasi, Rubens Maciel, André Luiz Jardini, Laís Pellizzer Gabriel

**Affiliations:** 1 Faculdade de Ciências Aplicadas, Universidade Estadual de Campinas, Limeira, SP, Brazil; 2 Eva Scientific Ltda, São Paulo, SP, Brazil; 3 Instituto Nacional de Ciência e Tecnologia em Biofabricação, Campinas, SP, Brazil

**Keywords:** Biopolymers, Bioreactors, Heart transplantation, Cardiomyoplasty, Cardiovascular diseases, Tissue engineering, Biopolímeros, Reatores biológicos, Transplante de coração, Cardiomioplastia, Doenças cardiovasculares, Engenharia tecidual

## Abstract

Cardiovascular diseases are the major cause of death worldwide. The heart has limited capacity of regeneration, therefore, transplantation is the only solution in some cases despite presenting many disadvantages. Tissue engineering has been considered the ideal strategy for regenerative medicine in cardiology. It is an interdisciplinary field combining many techniques that aim to maintain, regenerate or replace a tissue or organ. The main approach of cardiac tissue engineering is to create cardiac grafts, either whole heart substitutes or tissues that can be efficiently implanted in the organism, regenerating the tissue and giving rise to a fully functional heart, without causing side effects, such as immunogenicity. In this review, we systematically present and compare the techniques that have drawn the most attention in this field and that generally have focused on four important issues: the scaffold material selection, the scaffold material production, cellular selection and *in vitro* cell culture. Many studies used several techniques that are herein presented, including biopolymers, decellularization and bioreactors, and made significant advances, either seeking a graft or an entire bioartificial heart. However, much work remains to better understand and improve existing techniques, to develop robust, efficient and efficacious methods.

## RESUMO

Doenças cardiovasculares são responsáveis pelo maior número de mortes no mundo. O coração possui capacidade de regeneração limitada, e o transplante, por consequência, representa a única solução em alguns casos, apresentando várias desvantagens. A engenharia de tecidos tem sido considerada a estratégia ideal para a medicina cardíaca regenerativa. Trata-se de uma área interdisciplinar, que combina muitas técnicas as quais buscam manter, regenerar ou substituir um tecido ou órgão. A abordagem principal da engenharia de tecidos cardíacos é criar enxertos cardíacos, sejam substitutos do coração inteiro ou de tecidos que podem ser implantados de forma eficiente no organismo, regenerando o tecido e dando origem a um coração completamente funcional, sem desencadear efeitos colaterais, como imunogenicidade. Nesta revisão, apresentase e compara-se sistematicamente as técnicas que ganharam mais atenção nesta área e que geralmente focam em quatro assuntos importantes: seleção do material a ser utilizado como enxerto, produção do material, seleção das células e cultura de células *in vitro.* Muitos estudos, fazendo uso de várias das técnicas aqui apresentadas, incluindo biopolímeros, descelularização e biorreatores, têm apresentado avanços significativos, seja para obter um enxerto ou um coração bioartifical inteiro. No entanto, ainda resta um grande esforço para entender e melhorar as técnicas existentes, para desenvolver métodos robustos, eficientes e eficazes.

## INTRODUCTION

Cardiovascular diseases are the leading cause of death in the world. In 2015, World Health Organization (WHO) estimated that 17.7 million people died from cardiovascular diseases, accounting for 31% of deaths worldwide.^(^ ^1^ ^)^ A relevant disease in this scenario is acute myocardial infarction, resulting from insufficient transport of blood to the heart, caused mainly by coronary heart disease. Myocardial infarction leads to ventricular remodeling, fibrosis, necrosis, heart failure, among others, which may cause partial or total cardiac dysfunction.^(^ ^2^ ^)^ Considering the several disadvantages of heart transplantation, which is still the best option for patients with end-stage heart failure,^(^ ^3^ ^)^ and the restricted regenerative abilities of cardiomyocytes to heal the heart after an acute acute myocardial infarction,^(^ ^4^ ^)^ many studies in regenerative medicine have been performed creating alternatives for myocardial regeneration through tissue engineering.

Tissue engineering is a set of biomedical, biotechnological and engineering techniques that aim to maintain, regenerate or replace tissues or organs. Advances in tissue engineering are evident, and the application of this technology to the regeneration of myocardium has been increasingly explored, and presented encouraging results. The main approach of this scientific field is the creation of scaffolds, which contain cells that can be applied as cardiac grafts in the body, to have the desired recovery.

This review briefly presents the most widely used techniques in cardiac tissue engineering spanning two decades: from the late 1990s, when this tissue engineering application saw its first studies, to nowadays, when grafts with a broad potential for cardiac regeneration are sought.

## CARDIAC GRAFTS

**Figure 1:**
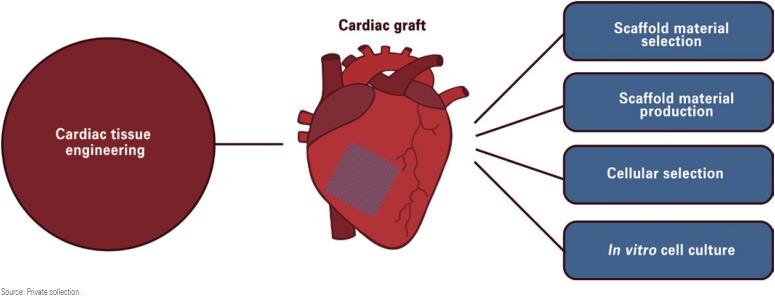
Cardiac graft engineering techniques

Source: Private collection.

The techniques used to obtain cardiac grafts focus on four important issues (Figure 1): (1) scaffold material selection; (2) scaffold material production; (3) cell selection; and (4) *in vitro* cell culture.

### Scaffold material selection

Biomaterials have been the focus of materials for use in tissue engineering, as traditional biomaterials or development of tissue engineering-specific variants. The approach usually taken into account is since biomaterials are able to positively interact with biological systems, they should then seek to improve the regeneration of the damaged tissue or effectively replacing it.

One of the most important classes of biomaterials comprises polymers, which are available with different compositions and properties. Polymers are the most used biomaterials for cardiac regeneration, and this class of materials may be divided into synthetic and natural polymers, in addition to synthetic/natural combination materials.^(^ ^5^ ^)^

Some synthetic biopolymers that have been used for myocardial tissue engineering include polyglycolic acid (PGA),^(^ ^6^ ^)^ polylactic-l-acid (PLLA), polylactic glycolic acid (PLGA) and polyurethane. This review focuses on the application of polyurethane in cardiac tissue engineering, considering that this biopolymer is one of the most used.

There is a variety of biomedical applications for PU, from durable devices to biodegradable scaffolds.^(^ ^7^ ^,^ ^8^ ^)^ Considering its good tissue and blood compatibility, cell adhesion and ductility properties,^(^ ^9^ ^)^ polyurethane has been investigated as an alternative for vascular grafts^(^ ^10^ ^)^ and other medical devices. However, long-term biostability has proved to be an obstacle for this type of application.^(^ ^10^ ^)^

**Figure 2:**
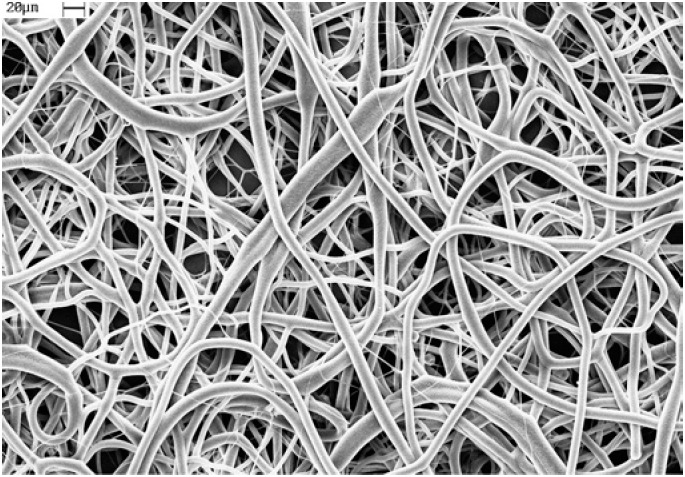
Scanning electron microscopy of electrospun polyurethane-based scaffold. Scale bar: 20*μ*m

In contrast, cardiac tissue engineering strategies focus on temporary polymer scaffolds with adjustable degradation rates, good porosity, biocompatibility and elastomeric properties, which can mechanically favor the tissue contraction inherent to the cardiac function. These properties are met in polyurethane-based scaffolds^(^ ^11^ ^)^(Figure 2), and different approaches using polyurethane have therefore been investigated, demonstrating the different possibilities and the versatility of polyurethane as a material for porous scaffolds in myocardial regeneration. Fujimoto et al., published a successful animal trial using a biodegradable, porous polyurethane heart patch that promoted a contractile phenotype, smooth muscle tissue formation and improved cardiac remodeling and contractile function at the chronic stage.^(^ ^12^ ^)^ Baheiraei et al., demonstrated the synthesis of a novel conductive, biodegradable polyurethane containing oligoaniline, as an electroactive conductive polymer in cell culture experiments.^(^ ^13^ ^)^

Natural polymers for scaffolds are inspired by the extracellular matrix (ECM) that holds the cells together in a native tissue. Therefore, some materials, such as collagen (mainly type I and III are found in the heart) and fibrin, have been extensively investigated in cardiac tissue engineering for their properties of natural interaction with cells.^(^ ^14^ ^,^ ^15^ ^)^ However, the mechanical properties of these materials, depending on the conformation in the form of gels or more solid structures, can be poor and not compatible with the cardiac tissue.^(^ ^5^ ^)^ One recent strategy to obtain natural polymers with a suitable size and shape (anatomy) is decellularization.

Considering the often poor mechanical characteristics of natural polymers, the combination of synthetic and natural polymers has been proposed to improve the weakness of each material to create a scaffold with better properties. Alperin et al., showed that embryonic stem cell-derived cardiomyocytes could be seeded on polyurethane films coated with collagen IV and laminin, demonstrating a higher number of contracting films than polyurethane without coating.^(^ ^16^ ^)^ Hong et al., generated a biohybrid composite combining ECM with polyurethane to improve bioactivity *in vivo,* and employed an electrospinning/electrospray method.^(^ ^17^ ^)^ Therefore, a promising approach for cardiac regeneration can be, for example, the synthesis of collagen-based polyurethane.

### Scaffold material production

An important issue that has been the object of investigation for groups involved in tissue engineering of the myocardium is the delivery method of the cells to the damaged site. One of the first technologies developed for cardiac regeneration was cellular cardiomyoplasty. This technology was very important for the study of cell types, their application and effects in cardiac regeneration. However, the delivery methods of the cells in the myocardial tissue used in this technology, such as the transvenous, endomyocardial and intracoronary routes, have not proven themselves satisfactory and had many disadvantages leading to inefficiencies.^(^ ^18^ ^)^ Thus, other cardiac tissue engineering strategies have been planned to improve the results of cardiac regeneration, including injectable biomaterials containing cells,^(^ ^19^ ^)^ and the creation of two or three-dimensional porous structures (patch or scaffold).

Many techniques have been investigated in order to create grafts to be implanted in the heart, and include fiber production methods, such as electrospinning^(^ ^7^ ^,^ ^8^ ^,^ ^20^ ^)^ and rotary-jet spinning,^(^ ^21^ ^)^ as well as cell-sheet engineering.^(^ ^22^ ^)^ Besides that, the most interesting and recent approaches are techniques, such as decellularization, aiming to obtain three-dimensional structures that not only may regenerate the existing heart, but be used to create an entire bioartificial organ.

**Figure 3:**
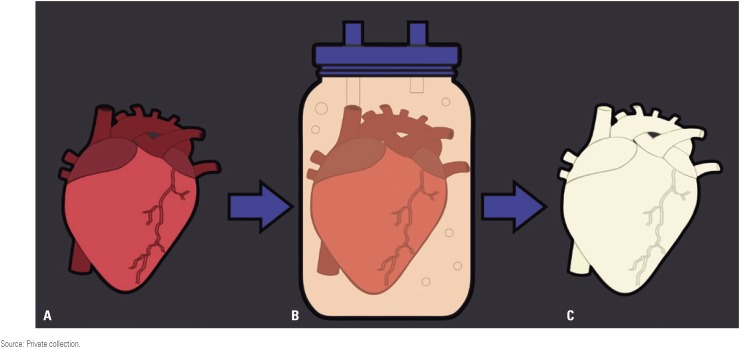
Decellularization schematic. (A) whole heart (may be human, *e.g.* cadaveric or a transplant reject or, more commonly, from an animal donor of suitable size/ anatomy match, often porcine) is placed into (B) an organ chamber of a decellularization bioreactor and appropriately connected up with suitable tubing and cannulae for perfusion, and the decellularization process initiated. Over a period of time, usually 1 or more days of continuous application of decellularization solution, the heart gradually whitens, indicative of the cell constituent of the tissue being washed away, largely leaving behind the collagen and other connective tissue substance and preserving to a great extent the original organs anatomical architecture with respect to vasculature and parenchyma (C)

Source: Private collection.

Decellularization is a process that consists of removing all cells from tissues or organs and maintaining the ECM intact (Figure 3), through different physical, chemical and enzymatic methods. This technique is widely used to obtain biologic scaffolds for clinical applications. The process of perfusion decellularization has been shown to be efficient in preserving the three-dimensional geometry of organs while eliminating the cells with a more even distribution of decellularization agents.^(^ ^23^ ^,^ ^24^ ^)^ This technique has been the most widely used for whole heart bioengineering, owing in part to the anatomical complexity of the macro and microanatomy of the heart organ, difficult to reproduce in detail by 100% synthetic means, but reasonably feasible through the decellularization approach. The choice of conduit for the perfusate is also important, and different conduits of vascular or parenchymal nature are viable alternatives for certain organs (*e.g.,* kidney-vascular: renal artery, renal vein; parenchymal: ureter), but for heart decellularization, the vascular perfusion route is preferred, presenting promising results.^(^ ^24^ ^)^

### Cellular selection

The cellular cardiomyoplasty technology is based on cell transplantation, consisting of supplying cells to the injured myocardial tissue aiming for the regeneration of the cardiac function that has been compromised.^(^ ^25^ ^)^ Many cell types have been used for cell transplantation into injured myocardium and are the same used as candidates in other myocardial tissue engineering approaches, including adult, fetal and neonatal cardiomyocytes,^(^ ^26^ ^,^ ^27^ ^)^ skeletal myoblasts,^(^ ^28^ ^)^ bone marrow derived stem cells (such as mesenchymal, endothelial and hematopoietic stem/ progenitor cells),^(^ ^29^ ^)^ embryonic stem cells,^(^ ^30^ ^–^ ^33^ ^)^ smooth muscle cells,^(^ ^34^ ^,^ ^35^ ^)^ adipose tissue derived stem cells,^(^ ^36^ ^,^ ^37^ ^)^ cardiac stem cells^(^ ^38^ ^)^ and the relatively novel technology of induced pluripotent stem cells (iPS cells).^(^ ^39^ ^,^ ^40^ ^)^ A variety of considerations must be made to choose the best cell type to be used in each situation, depending, *e.g.,* on its availability and convenience.^(^ ^41^ ^)^ As such, it is valuable to analyze the advantages and disadvantages of each type of cell (Table 1).

**Table 1:** Advantages and disadvantages of each type of cell used for cellular cardiomyoplasty

Cell type	Mechanism of action	Advantages	Disadvantages
Cardiomyocytes (adult, fetal and neonatal)	Myogenesis	Integration with the host tissue, as seen in rodents^(^ ^26^ ^,^ ^27^ ^)^ Adult cardiomyocytes' phenotype^(^ ^26^ ^)^	Ethics dilemma Inability to reproduce^(^ ^25^ ^)^ Limited availability Immunogenicity^(^ ^18^ ^)^ Short cell survival ^(^ ^25^ ^)^ Poor integration with host tissue in porcine^(^ ^27^ ^)^
Skeletal myoblasts	Myogenesis	Autologous High cell survival and engraftment^(^ ^42^ ^)^ Adult skeletal muscle phenotype^(^ ^28^ ^)^ Easy to isolate	High risk of arrhythmias^(^ ^32^ ^)^ Low structural and physiological integration with host tissue^(^ ^32^ ^)^
Bone marrow-derived stem cells (mesenchymal stem cells, endothelial progenitor cells, hematopoietic stem cells)		Multipotent Autologous Easy to isolate High expansion potential Presence of paracrine effects^(^ ^43^ ^)^	Possible angiogenesis at unwanted sites^(^ ^41^ ^)^ Limited availability
Mesenchymal stem cells	Myogenesis	Differentiate into cardiomyocytes-like phenotype^(^ ^44^ ^)^ Differentiation into endothelial cells as seen in canines^(^ ^45^ ^)^ Immunosuppressive properties^(^ ^46^ ^)^ High structural and physiological integration with host tissue^(^ ^43^ ^,^ ^45^ ^)^	Immunogenicity of allogenous mesenchymal stem cells^(^ ^47^ ^)^
Endothelial progenitor cells	Angiogenesis	Neovasculogenesis^(^ ^48^ ^)^	Limitations in number and migration of cells in patients with coronary artery disease^(^ ^49^ ^)^
Hematopoietic stem cells	Angiogenesis	Beneficial impact on left ventricular remodeling and angiogenesis^(^ ^50^ ^)^	Incapable of differentiating into cardiomyocytes^(^ ^50^ ^)^
Embryonic stem cells	Myogenesis	High proliferation^(^ ^51^ ^)^	Ethical dilemma
		Pluripotent^(^ ^51^ ^)^	Risk of teratoma formation^(^ ^51^ ^)^
		Differentiate into cardiomyocytes^(^ ^32^ ^)^	High immunogenicity^(^ ^52^ ^)^
		Integrate with host cardiomyocytes via gap junctions^(^ ^30^ ^)^ Can be pre-treated with pro-survival factors^(^ ^33^ ^)^	Limited availability
Smooth muscle cells	Myogenesis	Elastic properties improve heart function^(^ ^35^ ^)^	Immunogenicity^(^ ^34^ ^)^ Do not improve contractile function^(^ ^34^ ^)^
Adipose tissue-derived stem cells	Angiogenesis Myogenesis	Multipotent^(^ ^53^ ^)^ High availability^(^ ^36^ ^)^ Easy to isolate^(^ ^53^ ^)^ High proliferation^(^ ^36^ ^)^ Secrete growth factors^(^ ^37^ ^)^ Induce myogenesis and angiogenesis^(^ ^37^ ^)^	Poor long-term cell engraftment^(^ ^54^ ^)^ No significant differentiation^(^ ^55^ ^)^
Cardiac stem cells	Myogenesis	Autologous Multipotent^(^ ^38^ ^)^ Expand *in vitro* ^(^ ^38^ ^)^	Limited availability
Induced pluripotent stem cells	Myogenesis	Pluripotent Autologous Differentiate into cardiomyocytes^(^ ^39^ ^)^ Good availability High expansion potential	Risk of teratoma formation^(^ ^40^ ^)^

### *In vitro* cell culture

The next topic critical for creating a cardiac graft, following cellular selection, is the cell culture. From dishes to specialized cell culture facilities (bioreactors), research has been made to study how to promote cell proliferation, alignment, differentiation and maturation *in vitro,* before the implantation *in vivo.*

The potential for alignment of cells on the scaffold has been shown in some studies with polyurethane and *in vitro* cell culture in dishes. McDevitt et al., demonstrated that cardiomyocytes could be cultured on polyurethane films with printed laminin patterns, allowing the two-dimensional alignment of cells and presenting contractile response.^(^ ^11^ ^)^ Rockwood et al., prepared aligned and unaligned biodegradable polyurethane culture substrates using electrospinning, demonstrating that aligned scaffolds can generate a cellular organization similar to cardiac native tissue.^(^ ^20^ ^)^

To promote increased cellular proliferation, differentiation and maturation, the *in vitro* cell culture of the chosen cell types is performed in specialized cell culture facilities, be it in academia or industry. The standard infrastructure comprises clean rooms, carbon dioxide incubators, biological safety cabinets, sterile cell culture consumables, cell counters, and other standard equipment. In some cases,^(^ ^56^ ^)^ it may be warranted the use of cell bioreactors, based solely on the purpose of improving, refining and optimizing the quality and yield (expansion) of the cell itself. The objective of employing such cell bioreactors, that could include instruments such as wave bioreactors (GE Xuri, GE Healthcare, New York, NY, USA) and stirred tank bioreactors (Applikon, Delft, The Netherlands; Sartorius, Göttingen, Germany; and others) with microcarriers for adherent cell culture, or automated cell culture robots (SelecT & CompacT SelecT, Sartorius Stedim Biotech, Royston, United Kingdom; VANTAGE & STAR systems, Hamilton, Reno, NV, USA; Freedom EVO system, Tecan, Männedorf, Switzerland; Cytomat 10 system, Thermo Fisher, Waltham, MA, USA; and others) would be improving on the quality and yield feasibly attainable by traditional cell culture approaches. Looking more broadly on the instrument category as a whole, bioreactors may be described as systems with controlled conditions and parameters that enable the stimulation of cell growth or biotransformation of substrate into products of interest by living cells or its components, such as enzymes or organelles.^(^ ^56^ ^)^ Systems based on the production of bioproducts, such as proteins, lipids, among others, are called production bioreactors.^(^ ^57^ ^)^ Systems that instead focus on cell expansion and cell therapy, in order to have these cells as the output are called cell bioreactors.^(^ ^57^ ^)^ Lastly, systems used for tissue engineering, seeking to obtain mature tissue as an output, are called tissue bioreactors.^(^ ^57^ ^)^

Many types of bioreactors have been used for different applications in bioprocesses. The diversity of bioreactor design alternatives is based on specific parameters and conditions, such as heat or gas transfer and homogeneity, required for each application. Some examples of bioreactor designs are the stirred tank and the airlift reactors.^(^ ^58^ ^)^

The possibility of creating a dynamic environment with mechanical, physical and biochemical control makes tissue bioreactors a widely used technology in tissue engineering, due to the necessity of providing suitable stimuli for appropriate cellular differentiation and proliferation, and ECM properties for a tissue in development.^(^ ^59^ ^)^ Some studies in bioreactors for tissue engineering include bone,^(^ ^60^ ^)^ cartilage^(^ ^61^ ^)^ and cardiovascular system.^(^ ^62^ ^)^

**Figure 4:**
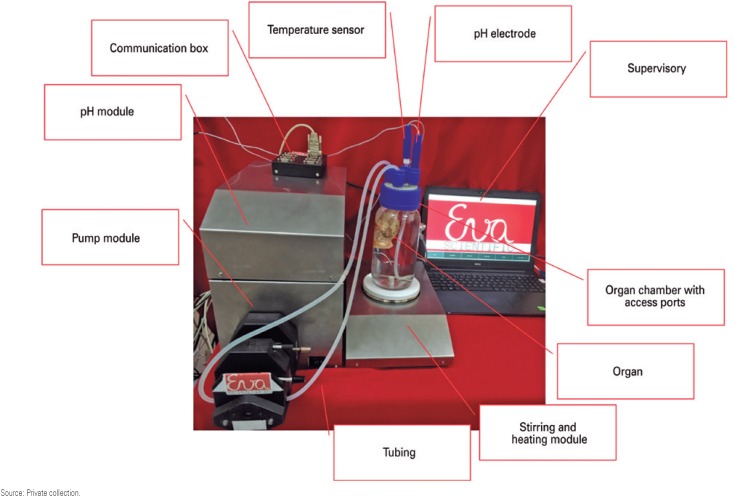
Eva Luxor™ bioreactor for tissues and organs with a trachea in the organ chamber, undergoing a decellularization process. The same bioreactor may be employed for tissue cultivation, using cell culture media instead of decellularization solutions (*e.g.,* detergents)

Source: Private collection.

Cardiovascular tissue engineering studies applying bioreactors involve blood vessels,^(^ ^63^ ^)^ heart valves^(^ ^57^ ^,^ ^64^ ^)^ and cardiac tissue culture.^(^ ^4^ ^,^ ^65^ ^–^ ^68^ ^)^ Cardiac tissue is extremely complex and bioreactors may help to better understand the influence of each parameter during the *in vitro* culture (Figure 4). Carrier et al., studied and characterized the effect of varying different parameters, such as cell source, cell seeding, flow and oxygen on cardiac tissue engineering structure and function, seeding well-mixed suspensions of cardiomyocytes in orbitally mixed dishes and spinner flasks.^(^ ^65^ ^)^ Bursac et al., demonstrated that three-dimensional cardiac muscle could be engineered using isolated cells and biodegradable polymer (PGA) scaffolds, on spinner flasks, to obtain specific structural and electrophysiological properties.^(^ ^6^ ^)^ Papadaki et al., showed the correlation between molecular, structural and electrophysiological properties, and how they can be improved using a high concentration of myocytes, laminin-coated surface-hydrolyzed PGA scaffolds, using rotating bioreactors and a low-serum medium.^(^ ^4^ ^)^ Carrier et al., also studied the effect of perfusion on improving the transport conditions of the culture, and creating constructs with relatively uniform spatial distributions of cardiac cells, using mixed flasks.^(^ ^66^ ^)^ Radisic et al., demonstrated that rapid gel-cell inoculation, followed by immediate perfusion, enabled fast and uniform seeding of cardiomyocytes in a high density whilst maintaining cell viability.^(^ ^67^ ^)^ Bursac et al., showed that neonatal rat cardiomyocytes cultured using rotating bioreactors in three-dimensional scaffolds had properties more similar to native tissue than cells cultured in monolayers.^(^ ^68^ ^)^ Gonen-Wadmany et al., studied the effect of a strain stimulation bioreactor to apply cyclic distension on engineered cardiac constructs, and improve cellular orientation *in vitro*.^(^ ^69^ ^)^ Lichtenberg et al., reported the development of a multifunctional bioreactor with four chambers and two separated compartments for three-dimensional co-cultivation of different cell types and culture conditions.^(^ ^70^ ^)^ As is apparent, many types of bioreactors have been designed for cardiac tissue engineering and the different approaches tested have paved the way for a large number of possible strategies, which may be improved, refined or optimized to obtain an ideal graft of mature myocardial tissue.

## CONCLUSIONS AND PERSPECTIVES

The heart is an extremely complex organ and the techniques influencing its regeneration depend on many variables of non-trivial character. These techniques generally focus on the scaffold material selection, scaffold material production, cellular selection and cellular cultivation *in vitro.* Many studies in this field have already made enormous progress, either looking for a graft or an entire bioartificial heart. However, much work remains to be done to better understand and solve the challenges of experimental technologies, improving on existing techniques and developing new techniques, protocols and methods.

For the material selection and structure, firstly, it is important to define the best material (synthetic, natural or hybrid) for cardiac applications. Some desired properties for these materials are adjustable degradation rates, good porosity, biocompatibility, hemocompatibility, good cell adhesion, mechanical and elastic properties compatible with the heart, and that the material permits an electrical coupling between cells and between the scaffold and the native tissue.^(^ ^11^ ^,^ ^13^ ^)^ Secondly, it is necessary to choose the technique to produce the scaffold in which cells are going to be seeded before the implantation. Future perspectives in this field focus on obtaining a scaffold from the threedimensional structure of an entire heart. In addition to decellularization, which is promising for cardiac tissue engineering application, another technology in vogue is three-dimensional bioprinting of tissues and organs.^(^ ^71^ ^)^ The combination of three-dimensional bioprinting, bioreactors and stem cells could provide for a novel enabling technology for the development of the next-generation human organ.

For cell selection and expansion *in vitro*, the first essential step is to determine the best cell type for the application (bone marrow-derived stem cells, cardiomyocytes, iPSC, among others), considering the availability and concerns about each cell type. After that, the cultivation of these cells *in vitro* is needed before seeding and subsequent tissue implantation. The most efficient technology to provide the proliferation and differentiation of these cells is the bioreactor. Many different types of bioreactors for cardiac tissue engineering applications have been studied, but it remains to be determined what approaches are the most suitable, with an ideal balance of advantages and disadvantages, appreciating that no single approach may tick all boxes.

Other opportunities for myocardial tissue engineering include finding the best combination of different techniques described herein, to achieve an ideal bioengineered myocardium for clinical applications and to study the influence of other aspects, such as the best period of time for implantation, with increased engraftment of cells at the recipient's tissue, after an acute myocardial infarction.^(^ ^18^ ^)^
